# Long‐term outcomes and toxicities of carbon‐ion radiotherapy in malignant tumors of the sphenoid sinus

**DOI:** 10.1002/hed.25965

**Published:** 2019-10-04

**Authors:** Yasuhito Hagiwara, Masashi Koto, Tapesh Bhattacharyya, Kazuhiko Hayashi, Hiroaki Ikawa, Kenji Nemoto, Hiroshi Tsuji

**Affiliations:** ^1^ Hospital of the National Institute of Radiological Sciences, National Institutes for Quantum and Radiological Sciences and Technology Chiba Japan; ^2^ Department of Radiation Oncology Faculty of Medicine, Yamagata University Yamagata Japan

**Keywords:** carbon‐ion radiotherapy, head and neck cancer, sphenoid sinus malignancy

## Abstract

**Background:**

Most of the primary sphenoid sinus tumors present with locally advanced stages with involvement of adjacent critical structures and are not amenable to radical resection. We sought to evaluate the safety and efficacy of carbon‐ion radiotherapy (C‐ion RT) for sphenoid sinus malignancies.

**Methods:**

This is a retrospective analysis of 22 patients of primary sphenoid carcinomas treated with definitive C‐ion RT.

**Results:**

Adenoid cystic carcinoma was the most common histology (15 patients, 68.2%). The median follow‐up of this cohort was 48.5 months. The actuarial local control and overall survival at 5 years were 51.0% and 62.7%, respectively. Grade 4 visual impairment and grade 4 brain necrosis were seen in six and one patient, respectively.

**Conclusion:**

C‐ion RT can provide a reasonably good clinical outcome in locally advanced sphenoid sinus malignancies with a marginally higher late toxicity profile because of extremely close proximity of the target volume to critical structures.

## 1 INTRODUCTION

Paranasal sinus tumors comprise of only around 2%‐3% of all head and neck cancers. Primary sphenoid sinus tumors are extremely rare and have been reported to occur in only 1%‐2% of all paranasal sinus tumors.^1^ As compared to other paranasal sinus tumors, they have a distinct clinical presentation of headache, diplopia, and various cranial neuropathies because of frequent skull base involvement.^2^, ^3^, ^4^, ^5^ Primary sphenoid sinus cancers are mostly epithelial in origin (squamous cell carcinomas [SCCs] and adenocarcinomas) but various other histologies such as adenoid cystic carcinomas, malignant melanomas, and sarcomas exist as well. The presence of air‐filled space permits the asymptomatic growth of those tumors until it invades the adjacent structures and poor anatomical access of sphenoid sinus makes the early diagnosis of those rare tumors difficult. Most of the patients present with symptomatic advanced disease involving adjacent critical structures. Those advanced sphenoid sinus tumors tend to have dismal prognosis compared with other paranasal sinus tumors.^6^ For nasal cavity and paranasal sinus tumors, the American Joint Committee on Cancer has defined a separate staging system for maxillary sinus and nasoethmoid complex for SCCs. The diagnosis and management of these tumors depend on interdisciplinary approach and it requires multimodality therapy to get the optimal treatment outcome.

Treating those tumors always remains technically challenging both to surgeons as well as to radiation oncologists because of its close proximity to critical structures. Although surgery is the mainstay of treatment for early primary sphenoid sinus malignancies however they present mostly in locally advanced stages and involves critical structures such as skull base and cranial nerves. Hence they are not suitable for R0 surgical resection most of the times and radiotherapy is often used as the upfront radical treatment to control those tumors. The primary obstacle to cure hypoxic radioresistant locally advanced tumors of sphenoid sinus with radiotherapy is to achieve escalated tumoricidal dose because of close proximity of target volume to neural structures and optic apparatus. Truong et al had already shown that proton therapy was feasible with excellent normal tissue sparing due to its unique physical property which allows sharp dose fall off between target volume and normal critical structures exploiting the Bragg peak effect.^7^

Carbon ion offers potentially superior dose distributions which allows to escalate the dose with the improved potential to spare the critical organs at risk (OAR). It also has higher linear energy transfer and increased relative biological effectiveness (RBE) leading to a possibility of increased tumor cell killing as compared to other radiation modalities such as photons or protons. Thinking of radioresistance of non‐SCCs of sphenoid sinus and the close proximity of the target volume to critical structures, carbon‐ion radiotherapy (C‐ion RT) seems to be a promising curable option for many inoperable primary non‐SCCs owing to its unique physical dose distribution and enhanced biological effectiveness.^8^, ^9^

We report here a retrospective analysis of patients with primary sphenoid sinus malignancies who were treated with C‐ion RT.

## 2 MATERIALS AND METHODS

This is a single center retrospective analysis of 22 patients of primary sphenoid malignancies who were treated with C‐ion RT between October 1995 and April 2017 at our institute. Patients provided informed consent authorizing the use of their personal information for research purposes. This study was approved by the Institutional Review Board of our institute and was carried out following the Declaration of Helsinki. This trial is registered with UMINCTR as UMIN000034858. Patients were diagnosed with primary sphenoid sinus malignancies if the tumor epicenter was found to be on the center of sphenoid sinus on imaging. All histopathology slides were reviewed by central pathologist before starting C‐ion RT. The inclusion criteria are as follows: (a) histologically proven malignancy, (b) medically inoperable cases or those who refused surgery, (c) treated with definitive intent, (d) nonmetastatic disease, and (e) Eastern Cooperative Oncology Group performance status 0‐2. Patients who received radiotherapy previously for the same disease were excluded from this study.

### 2.1 Study endpoints

The primary endpoints of this study were overall survival (OS) and local control. Local control was defined as no evidence of tumor regrowth within the planning target volume (PTV). Secondary end points acute and late toxicities were evaluated according to National Cancer Institute Common Terminology Criteria for adverse events version 4.0.

### 2.2 Treatment protocol

As the treatment table can be rotated allowing a greater range of beam angle flexibility, all patients were immobilized in a customized cradle (Moldcare, Alcare, Tokyo, Japan) with a relatively thicker shell (3 mm thickness) made of low‐temperature thermoplastic mask (Shellfitter, Kuraray, Osaka, Japan). A set of noncontrast CT images with 2.0‐2.5 mm slice thickness were obtained for treatment planning. Planning CT images were fused with contrast enhanced CT and gadolinium enhanced MRI images for accurate target volume delineation. Noncontrast simulation CT was used for treatment planning to avoid miscalculation of particle range and to reduce the dose uncertainties.

Gross tumor volume (GTV), clinical target volume (CTV), PTV and adjacent critical OAR were outlined. GTV was contoured based on macroscopic tumor visible on radiological imaging, nasal endoscopy, and clinical findings. A minimum of 5 mm margin was added to GTV to create CTV. CTV was further modified respecting the biology, natural history, and clinical course of the disease. Furthermore, 2 mm margin for setup error was added around CTV to create final PTV. Brainstem and optic chiasma dose constraints were maximum point dose (*D*_max_) of 30 Gy (RBE) and limiting dose for uninvolved optic nerve was *D*_max_ of 40 Gy (RBE). A limiting dose was not established for the brain. Three‐dimensional treatment planning was performed with HIPLAN software (National Institute of Radiological Sciences, Chiba, Japan) and Xio‐N (ELEKTA, Stockholm, Sweden; and Mitsubishi Electric, Tokyo, Japan). The dose prescription was at the isocenter and a minimum of 90% isodose line was planned to encompass the PTV. Treatment planning was performed with biological treatment plan optimization which took account a clinical RBE value of 3 at distal part of Bragg Peak. The prescribed dose was 52.8 Gy (RBE) or 64 Gy (RBE) in 16 fractions over 4 weeks.

During each fraction of C‐ion RT, patient's position was verified with a sophisticated computer aided online positioning system. After patient positioning with customized immobilization devices, digital orthogonal radiographs were acquired and transferred to the computerized positioning system. The setup images were compared with the reference images digitally reconstructed from radiographs. We allowed a setup error of maximum 2 mm in different directions.

One malignant melanoma patient received one cycle of concurrent and four cycles of adjuvant chemotherapy with a dose of Vincristine 0.7 mg/m^2^ day 1, Nimustine Hydrochloride 70 mg/m^2^ day 1, and DTIC 120 mg/m^2^ days 1‐5 repeated every 4 weeks. One patient of rhabdomyosarcoma received one cycle of neoadjuvant chemotherapy based on Vincristine, Cyclophosphamide, and Actinomycin D outside our institute.

### 2.3 Follow‐up

The clinical follow‐up was scheduled every 2 to 3 months for the first 2 years and 3‐6 months up to 5 years. After 5 years patients were followed up every 6 months. Most of the patients underwent contrast enhanced CT and MRI of the head and neck region at 3‐6 months intervals. Comprehensive clinical evaluation including nasal endoscopy was performed in each follow‐up.

### 2.4 Statistical analysis

The follow‐up time was calculated from the start date of C‐ion RT to the last date of follow‐up. Local control, OS, and progression‐free survival were calculated using Kaplan‐Meier method. For univariate analysis, log rank test was used to compare local control and OS among different subgroups based on patient, tumor, and treatment related factors such as age, sex, performance status, intracranial extension, median GTV, histology of the tumor, and dose fractionation. Here, T classification or composite staging was not included in univariate analysis because of lack of any particular staging system for primary sphenoid sinus malignancies. A *P* value <.05 was considered statistically significant. All statistical analyses were performed using R software, version 3.4.4.

## 3 RESULTS

### 3.1 Baseline characteristics

The baseline characteristics of the patients are shown in Table 1. The median age of this group of patients was 55 years (range 24‐77 years). Men to women ratio was 1.2 (12/10). The most common histology in this cohort was adenoid cystic carcinoma (15 patients 68.2%). The most common symptom at presentation was double vision (10 cases 45.5%) followed by facial pain (7 cases 31.8%). The clinical signs and symptoms at presentation are provided in Table 2. Median GTV was 59.5 cc (range 9‐120 cc). Most of the patients were treated with 57.6 Gy (RBE) or 64 Gy (RBE)/16 fractions/4 weeks (21 patients 95.4%).

**Table 1:** Baseline characteristics

Characteristics		Number (%)
Number of patients		22 (100)
Sex	Men/women	12 (54.5)/10 (45.5)
Age, years	Median/range	55/24‐77
Performance status	0/1	12 (54.5)/10 (45.5)
Histologic type	Adenoid cystic ca.	15 (68.2)
	Malignant melanoma	2 (9.1)
	Squamous cell ca.	2 (9.1)
	Mucoepithelial ca.	1 (4.5)
	Rhabdomyosarcoma	1 (4.5)
	Carcinoma	1 (4.5)
Temporal fossa involvement	No/Yes	6 (27.3/16 (72.7)
Intracranial involvement	No/Yes	4 (18.2)/18 (81.8)
Brain parenchyma involvement	No/Yes	15 (68.2)/7 (31.8)
Gross tumor volume, cc	Median/range	59.5/9–120
Carbon‐ion radiotherapy		
Fractionation	16 fractions /4 weeks	22 (100)
Total dose	52.8 Gy (RBE)	1 (4.5)
	57.6 Gy (RBE)	10 (45.5)
	64.0 Gy (RBE)	11 (50.0)
Beam delivery	Passive/spot scanning	17 (77.3)/5 (22.7)

Abbreviation: RBE, relative biological effectiveness.

**Table 2:** Clinical symptoms and signs

Signs and symptoms	Number (%)
Diplopia	10 (45.5)
Facial pain	7 (31.8)
Facial numbness	4 (18.1)
Nasal bleeding	6 (27.2)
Dimness of vision	5 (22.7)
Nasal obstruction	3 (13.6)
Headache	2 (9.1)
Proptosis	1 (4.5)

### 3.2 Treatment outcome

**Figure 1:**
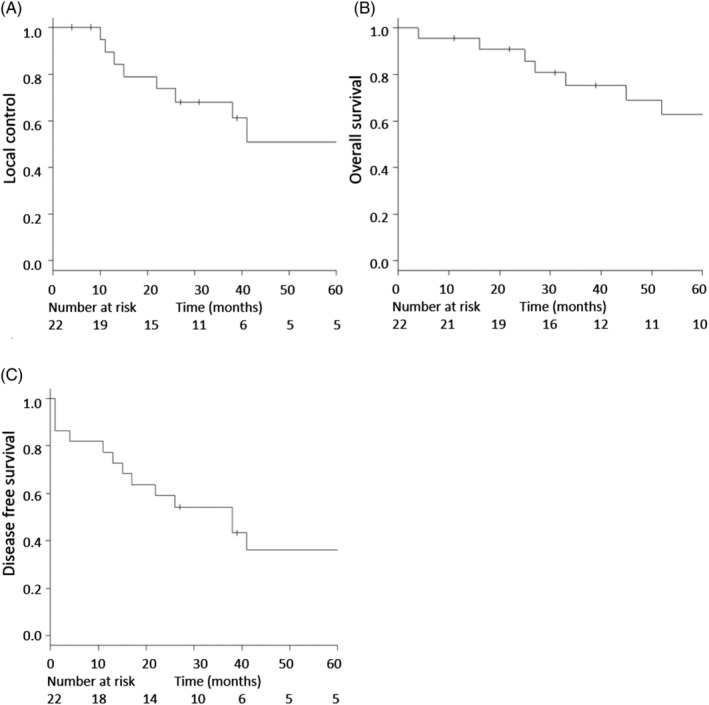
A, Local control; B, overall survival; and C, disease‐free survival in the patients cohort (number of patients = 22)

The median follow‐up period of all the patients and only surviving patients in this study were 48.5 months (range of 3‐133 months) and 70 months (range of 11‐133 months), respectively. The actuarial local control at 2, 3, and 5 years were 73.7% (95% confidence interval [CI]: 47.9‐88.1), 68.0% (95% CI: 42.1‐84.2), and 51.0% (95% CI: 23.4‐73.2), respectively. The OS at 2, 3, and 5 years were 90.7% (95% CI: 67.6‐97.6), 75.2% (95% CI: 50.2‐88.9), and 62.7% (95% CI: 36.7‐80.5), respectively. The 2, 3, and 5 years disease‐free survival (DFS) in this cohort were 59.1% (95% CI: 36.1‐76.2), 54.2% (95% CI: 31.6‐72.2), and 36.1% (95% CI: 15.6‐57.2), respectively. Local control, OS, and DFS are shown in Figure 1.

### 3.3 Patterns of failure

A total of 14 patients developed recurrence. Median time to overall recurrence was 27 months. Out of those 14 patients, 9 patients developed local recurrence and 7 patients developed distant metastasis. Among nine patients with local recurrences, six patients had central recurrences and three patients developed marginal recurrences. Two of them developed local recurrence and distant metastasis simultaneously. The lung was the most common site of distant metastasis (four patients) followed by the bone (two patients), the liver (one patient), and the carcinomatous meningitis in one patient. One patient had synchronous bone and lung metastasis. Median time to local failure and distant metastasis was 22 and 4 months, respectively. None of the patients developed regional recurrence.

### 3.4 Analysis of prognostic factors

Univariate analysis was used to compare local control and OS among different variables age, sex, pathology, involvement of temporal fossa, intracranial extension, involvement of brain parenchyma, median GTV, the minimum dose to 1 cc volumes (Dmin1cc) of GTV, the minimum dose to 0.1 cc volumes (Dmin0.1cc) of GTV, total dose, and type of beam delivery (Table 3). On univariate analysis, patients with adenoid cystic histology have shown better OS as compared to other histologies (4 year OS 81.5% vs 42.9%; *P* = .04). Patients with temporal fossa involvement had a higher propensity to develop local recurrence although statistically significant association could not be found out (4‐year local control 100.0% vs 36.7%; *P* = .05). On dose volume histogram (DVH) analysis patients with GTV Dmin0.1cc receiving more than 52 Gy (RBE) had a trend toward better local control and OS but could not reach statistical significance.

**Table 3:** Univariate analysis of different prognostic variables

Prognostic variables	Category	No. of patients	4‐year OS (%)	*P* value	4‐year local control (%)	*P* value
Age	<55 years	11	70.7	.88	62.5	.58
	≥55 years	11	68.6		38.2	
Sex	Men	12	58.3	.56	40.0	.69
	Women	10	77.8		62.2	
Histology	ACC	15	81.5	.04	46.4	.68
	Others	7	42.9		60.0	
Temporal fossa involvement	No	6	62.5	.84	100.0	.05
	Yes	16	71.5		36.7	
Intracranial involvement	No	4	75.0	.62	75.0	.97
	Yes	18	68.3		43.3	
Brain parenchyma involvement	No	15	64.2	.83	47.5	.56
	Yes	7	80.0		66.7	
Median GTV (cc)	<60	11	61.4	.73	45.7	.86
	≥60	11	75		60.0	
GTV Dmin1cc (Gy [RBE])	<56	12	63.5	.32	51.4	.97
	≥56	10	76.2		53.3	
GTV Dmin0.1cc (Gy [RBE])	<52	11	56.1	.17	NA	.11
	≥52	11	80.0		66.7	
Total dose (Gy [RBE])	<64	11	57.1	.35	35.4	.12
	≥64	11	80.8		70.0	
Beam type	Passive	17	70.6	.29	66.7	.77
	Scanning	5	100		75.0	

Abbreviations: Dmin0.1cc, minimal dose to 0.1 cc volumes; Dmin1cc, minimal dose to 1 cc volumes; GTV, gross tumor volume; NA, not available; OS, overall survival; RBE, relative biological effectiveness.

### 3.5 Acute and late toxicities

All patients tolerated C‐ion RT and could complete the treatment without any break. Grade 2 oral mucositis was seen in 13 patients (59.1%) and only 1 patient had grade 2 dermatitis. None of the patients developed CTCAE grade 3 or higher acute toxicities.

**Figure 2:**
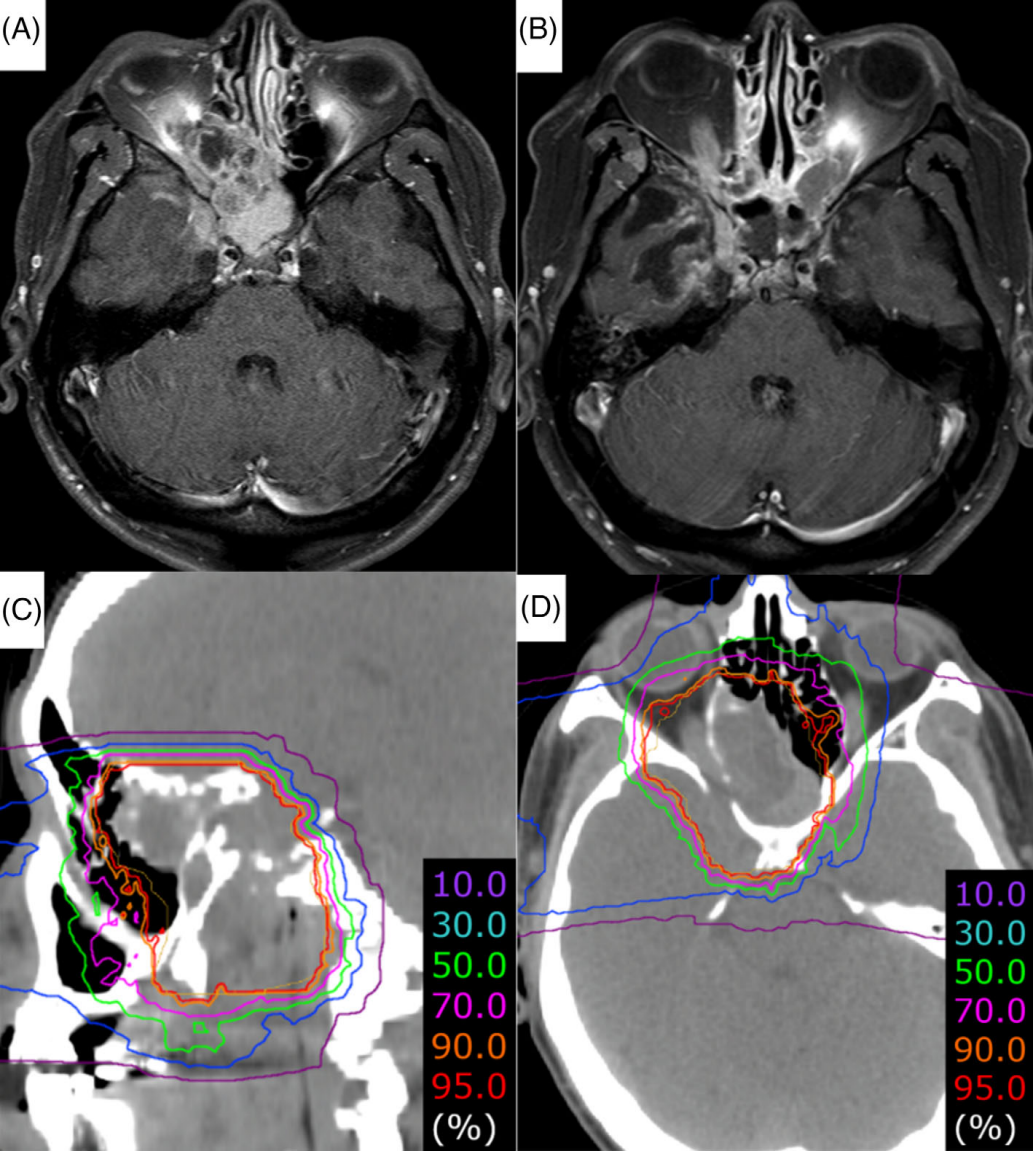
A representative case of the primary malignant tumor of sphenoid sinus. The histology of the sphenoid sinus tumor was confirmed as adenoid cystic carcinoma. The tumor invaded the intracranial fossa, right cavernous sinus, apex of orbit, nasopharynx, and maxilla along the maxillary division of the trigeminal nerve. A total dose of 64 Gy (RBE) was delivered to the tumor in 16 fractions. There is no evidence of disease after 10 years of carbon‐ion radiotherapy. However, the patient developed grade 4 right visual impairment and grade 2 brain necrosis with mild clinical symptoms of radiation‐induced right temporal lobe injury simultaneously at 3 years and 6 months after carbon‐ion radiotherapy. A, A gadolinium‐enhanced T1 axial MRI of sphenoid sinus before carbon‐ion radiotherapy. B, A gadolinium‐enhanced T1 axial MRI after 10 years of carbon‐ion radiotherapy. C, Dose distribution of carbon‐ion radiotherapy (sagittal view). D, Dose distribution of carbon‐ion radiotherapy (axial view) [Color figure can be viewed at http://wileyonlinelibrary.com]

Out of 22 patients, 20 (90.9%) had tumor invasion toward optic canal. Of the 20 patients, 10 (45.5%) had tumor touch or involvement into optic nerve and 5 (22.7%) had obvious dimness of vision before C‐ion RT. Grade 4 ipsilateral visual impairment was seen in six patients (27.2%). The median time to develop grade 4 visual impairment was 47 months (range 19‐71 months). Two patients (9.1%) developed grade 3 brain necrosis and both presented with seizures which was well controlled with antiepileptic drugs. The median time to develop grade 3 brain necrosis was 61 months. Grade 4 brain necrosis was seen in one patient which was complicated by abscess formation with superimposed infection and required emergency surgical intervention. Out of those three patients who developed grades 3 and 4 brain necrosis, two already had intracranial extension before C‐ion RT. A representative case is shown in Figure 2.

## 4 DISCUSSION

Primary sphenoid sinus tumor itself is extremely rare; reports of radiotherapy in this setting are further rarer in literature; this retrospective analysis is going to be the first report evaluating the outcome of C‐ion RT in those rare tumors.

Primary sphenoid sinus cancers like other sinonasal cancers are mainly epithelial in origin (SCCs or adenocarcinomas). Truong et al treated primary sphenoid carcinomas with proton out of which 50% were of SCCs.^7^ In a study by Hoppe et al, 49% of sinonasal carcinomas were of SCCs.^10^ Blanco et al included only SCCs (82.1%) and adenocarcinomas (17.9%) in their study.^11^ The index study is unique as we are going to report first‐hand experience on primary sphenoid tumors with predominantly non‐SCC histologies (adenoid cystic carcinoma 68%, malignant melanoma 9%) with C‐ion RT. From two decades of our C‐ion RT experience in head and neck cancer, SCCs are not the preferred candidates for C‐ion RT. As SCCs are relatively radio‐ and chemosensitive, C‐ion RT may not offer incremental benefit as compared to chemoradiation in these tumors. Carbon ion can be used in selected cases of SCC in which chemoradiation is contraindicated because of medical comorbidities such as renal impairment or poor general condition, and OAR constraints cannot be achieved with intensity modulated radiotherapy (IMRT). In the present study, two patients of SCC were included as they had refused photon‐based chemoradiation and preferred C‐ion RT.

Patients with cancer of the paranasal sinuses have been shown to have excellent outcomes when treated with a multimodality approach including surgery and radiotherapy. As sphenoid sinus cancers present in the locally advanced stages and are very close to OAR, practically it becomes very difficult to routinely adopt the optimal approach. In our study cohort, all the patients were treated with definitive C‐ion RT which makes it exceedingly difficult to compare our outcome with other studies where most of the patients were of SCC histology, and a majority of them underwent photon‐ or proton‐based radiotherapy as adjuvant treatment after primary surgery. Truong et al in their proton therapy experience revealed that 50% of the patients had SCC followed by adenoid cystic carcinomas in 35% of cases. Thirty‐five percent patients underwent partial resection of tumors prior to proton therapy in the form of craniofacial resection, transfacial resection, or endoscopic approach. The remaining 65% of the patients underwent biopsy alone. The 2 years locoregional control, DFS and OS were 86%, 31%, and 53%, respectively.^7^ In GETTEC study by Vedrine et al, out of 23 patients of primary sphenoid malignancies, 10 patients underwent surgery. Definitive or adjuvant conformal radiotherapy was performed in 18 patients. They had extremely varied histologies with lymphoma being the most common (21.7%), followed by adenoid cystic carcinomas (17.4%), and median locoregional control was 22 months.^12^ In our cohort, the actuarial local control rates at 2, 3, and 5 years were 74%, 68%, and 51% respectively. The OS rates at 2, 3, and 5 years were 91%, 75%, and 63% respectively. In the definitive setting and especially thinking of radioresistant locally advanced unresectable non‐SCC histologies of sphenoid sinus region, our study showed excellent results with C‐ion RT. Comparison with other series of primary sphenoid carcinomas are shown in Table 4.

**Table 4:** Comparison with other series of primary sphenoid tumors

Author	No. of patients	Predominant histology	Treatment modality	Local control	OS
Truong et al 2009^6^	20	SCC (50%)	Surgery and proton therapy	86% at 2 years (locoregional control)	53% at 2 years
De Monte et al 2000^1^	27	SCC (33.3%) Adenoid cystic carcinoma (14.8%)	Surgery, radiotherapy, and chemotherapy in different combinations	Seven out of 27 patients had no evidence of disease	For SCC 44% at 2 years 30% at 5 years
Vedrine et al 2009^14^	23	Lymphoma (21.7%) Adenoid cystic carcinoma (17.4%)	Surgery, radiotherapy, and chemotherapy in different combinations	Median locoregional control 22 months	50% at 5 year
Current study	22	Adenoid cystic carcinoma (68%)	C‐ion RT	74% at 2 years 51% at 5 years	91% at 2 years 63% at 5 years

Abbreviations: C‐ion RT, carbon‐ion radiotherapy; OS, overall survival; SCC, squamous cell carcinoma.

Koto et al in their multicentric Japan Carbon‐ion Radiation Oncology Study Group study evaluated the efficacy and safety of C‐ion RT in locally advanced sinonasal malignant tumors in which 48% of the cases were of malignant melanoma and 26% had adenoid cystic carcinomas. Apart from the nasal cavity, majority of the patients had maxillary sinus and ethmoid sinus involvement (40%). Only 2% of the cases had sphenoid primary. The 2 and 5 years local control rates were 84.1% and 71.2%, respectively.^8^ One of the possible explanation of achieving less local control in the current study as compared to previous C‐ion RT report of sinonasal cancers^8^ can be suboptimal coverage of target volume because of close proximity of target volume to critical OARs. It could be substantiated by the fact that patients who received GTV Dmin0.1cc ≥ 52Gy (RBE) (n = 11) had shown a trend toward improved local control as compared to patients with GTV Dmin0.1cc < 52 Gy (RBE) (n = 11), although it was not statistically significant. In this study, median GTV dose, GTV Dmin1cc, and GTV Dmin0.1cc were evaluated to find out the cold spot or any under coverage within GTV. Absolute minimal dose to fixed volumes was considered more appropriate as compared to generalize a dose that covered 95% (D95%) or D98% parameters expressed in relative terms. Increase in dose coverage to the target can improve the clinical outcomes further. Total dose is difficult to escalate further because of critical location of the target volume and its close proximity to critical structures, which will lead to increased late toxicities. We believe that better dose coverage of the target and sparing of OARs will be possible with increasing use of integrated spot scanning and rotating gantry with wider angle flexibility.

Truong et al found oropharynx and anterior cranial fossa involvement to be significant predictive factors for DFS, whereas only invasion of brain parenchyma was predictive of inferior OS.^7^ In our cohort, majority of the patients had adenoid cystic carcinomas in which local invasion to adjacent cranial nerves and high propensity of perineural spread across skull base lead to temporal fossa involvement in more than 70% of cases. When we tried to analyze the disease outcome according to tumor extension, patients with temporal fossa involvement (16cases) had a trend toward poorer local control than the patients without temporal fossa involvement (6cases) although it could not reach statistical significance (4 year local control rate 34.3% vs 100%; *P* = .05). Both the series pointed out one point in common, that intracranial involvement is associated with poor prognosis and it should be proposed to be incorporated in the future staging system of primary sphenoid sinus tumor.

In the present study, brain injury and dimness of vision were common late toxicities. In our cohort, 18 patients had intracranial involvement and 7 of them had brain parenchyma involvement prior to C‐ion RT. Grade 3 or more brain injury was developed in three patients (13.6%) out of which two patients already had intracranial extension before starting C‐ion RT. Grade 3 brain injury of those patients could be easily managed with conservative approach. In those patients, it was difficult to achieve the brain constraints of V40 or V50 within the tolerance limit^13^ because of prior intracranial involvement. Integrating spot scanning, intensity modulated carbon ion therapy (IMCT), and use of rotating gantry device with multiple angle flexibilities will be of great help to optimize dose distributions and be of one of the possible solution.^14^ Large fraction size might be just one of the contributing factors in marginally higher radiation‐induced brain injury. Hence, improving dose distribution is more important than reducing the fraction size to reduce the late brain injuries further. None of our patients developed grade 5 brain injury or symptoms of CSF leakage.

Grade 4 ipsilateral visual impairment was seen in 27.2% of our patients, which was quite higher than the proton series by Truong et al^7^ or other IMRT series of sinonasal cancers.^15^, ^16^ In the present cohort, most of the patients presented with locally advanced disease with tumor invasion toward optic canal in 90.9% cases, tumor just touching or involving the optic nerve in 45.5% cases which was the most critical factor for higher risk of visual impairment, and it also made difficult to keep the *D*_max_ of the optic pathway within the safest limit possible. Moreover, most of the cases (77.2%) were treated with passive beam delivery systems with higher proximal dose. Integrating spot scanning, IMCT and use of rotating gantry device may be useful to improve the dose conformity and reduce the treatment‐related morbidity.^14^ GTVs in our cohort were much larger than the postoperative residual tumor volumes of other IMRT or particle therapy series which made it technically challenging to cover the target volume with optimal dose sparing the optic apparatus. We also conducted a long follow‐up of surviving patients for 70 months, therefore, allowing more time for late toxicities to develop. Rates of toxicity are difficult to compare across studies because of the heterogeneity of tumor locations, stages, histologies, treatment modalities, and moreover, very few studies exist in primary sphenoid tumors.

Several points need to be noted. It was a retrospective study with a small sample size and therefore it has inherent bias. The patient population was quite heterogenous in terms of histology. Owing to the rarity of the disease, it is difficult to conduct prospective studies and have to rely on retrospective series. This is not only the first series of C‐ion RT in primary sphenoid tumors, it also shows the contribution of C‐ion RT in dealing radioresistant histologies in such critical locations.

## 5 CONCLUSION

Treating locally advanced unresectable primary sphenoid sinus tumors always remained technically challenging. C‐ion RT offers reasonably good clinical outcome in locally advanced primary sphenoid sinus tumors, especially in radioresistant histologies with an expense of marginally higher >grade 3 late toxicities because of extremely close proximity to critical structures. However, we hope that integrating enhanced use of spot scanning, IMCT, and rotating gantry will reduce the treatment‐related late morbidities and improve the outcomes in future.

## CONFLICT OF INTEREST

The authors declared no conflict of interest.

## References

[1] DeMonte F , Ginsberg LE , Clayman GL . Primary malignant tumors of the sphenoidal sinus. Neurosurgery. 2000;46:1084‐1091. 10807240 10.1097/00006123-200005000-00012

[2] Alexander FW . Primary tumors of the sphenoid sinus. Laryngoscope. 1963;73:537‐546. 14011951 10.1288/00005537-196305000-00006

[3] Harbison JW , Lessell S , Selhorst JB . Neuro‐ophthalmology of sphenoid sinus carcinoma. Brain. 1984;107:855‐870. 6478180 10.1093/brain/107.3.855

[4] Lawson W , Reino AJ . Isolated sphenoid sinus disease: an analysis of 132 cases. Laryngoscope. 1997;107:1590‐1595. 9396670 10.1097/00005537-199712000-00003

[5] Levine H . The sphenoid sinus, the neglected nasal sinus. Arch Otolaryngol. 1978;104:585‐587. 697636 10.1001/archotol.1978.00790100039008

[6] Dulguerov P , Jacobsen MS , Allal AS , Lehmann W , Calcaterra T . Nasal and paranasal sinus carcinoma: are we making progress? A series of 220 patients and a systematic review. Cancer. 2001;92:3012‐3029. 11753979 10.1002/1097-0142(20011215)92:12<3012::aid-cncr10131>3.0.co;2-e

[7] Truong MT , Kamat UR , Liebsch N , et al. Proton radiation therapy for primary sphenoid sinus malignancies: treatment outcome and prognostic factors. Head Neck. 2009;31:1297‐1308. 19536762 10.1002/hed.21092

[8] Koto M , Demizu Y , Saitoh J , et al. Definitive carbon‐ion radiation therapy for locally advanced sinonasal malignant tumors: subgroup analysis of a multicenter study by the Japan Carbon‐Ion Radiation Oncology Study Group (J‐CROS). Int J Radiation Oncol Biol Phys. 2018;102:353‐361. 10.1016/j.ijrobp.2018.05.074 30191869

[9] Sulaiman NS , Demizu Y , Koto M , et al. Multicenter study of carbon‐ion radiation therapy for adenoid cystic carcinoma of the head and neck: subanalysis of the Japan Carbon‐Ion Radiation Oncology Study Group (J‐CROS) study (1402 HN). Int J Radiat Oncol Biol Phys. 2018;100:639‐646. 29413278 10.1016/j.ijrobp.2017.11.010

[10] Hoppe BS , Stegman LD , Zelefsky MJ , et al. Treatment of nasal cavity and paranasal sinus cancer with modern radiotherapy techniques in the postoperative setting the MSKCC experience. Int J Radiat Oncol Biol Phys. 2007;67:691‐702. 17161557 10.1016/j.ijrobp.2006.09.023

[11] Blanco AI , Chao KS , Ozyigit G , et al. Carcinoma of paranasal sinuses: long‐term outcomes with radiotherapy. Int J Radiat Oncol Biol Phys. 2004;59:51‐58. 15093898 10.1016/j.ijrobp.2003.09.101

[12] Vedrine PO , Thariat J , Merrot O , et al. Primary cancer of the sphenoid sinus—a GETTEC study. Head Neck. 2009;31:388‐397. 18972425 10.1002/hed.20966

[13] Koto M , Hasegawa A , Takagi R , et al. Risk factors for brain injury after carbon ion radiotherapy for skull base tumors. Radiother Oncol. 2014;111:25‐29. 24332023 10.1016/j.radonc.2013.11.005

[14] Mohamad O , Makishima H , Kamada T . Evolution of carbon ion radiotherapy at the National Institute of Radiological Sciences in Japan. Cancer. 2018;10:66. 10.3390/cancers10030066 PMC5876641 29509684

[15] Duprez F , Madani I , Morbe'e L , et al. IMRT for sinonasal tumors minimizes severe late ocular toxicity and preserves disease control and survival. Int J Radiat Oncol Biol Phys. 2012;83:252‐259. 22027259 10.1016/j.ijrobp.2011.06.1977

[16] Coombs SE , Konkel S , Schulz‐Ertner D , et al. Intensity modulated radiotherapy (IMRT) in patients with carcinomas of the paranasal sinuses: clinical benefit for complex shaped target volumes. Radiat Oncol. 2006;1:23. 16859556 10.1186/1748-717X-1-23 PMC1557519

